# Editorial: Latest Advances on Excitatory Synapse Biology

**DOI:** 10.3389/fnsyn.2021.768651

**Published:** 2021-09-28

**Authors:** Kimberly M. Huber, Pierre Paoletti, P. Jesper Sjöström

**Affiliations:** ^1^Department of Neuroscience, O'Donnell Brain Institute, University of Texas Southwestern Medical Center, Dallas, TX, United States; ^2^Institut de Biologie de l'Ecole Normale Supérieure (IBENS), Ecole Normale Supérieure, Université PSL, CNRS, INSERM, Paris, France; ^3^Centre for Research in Neuroscience, Brain Repair and Integrative Neuroscience Program, Department of Medicine, Department of Neurology and Neurosurgery, The Research Institute of the McGill University Health Centre, Montreal General Hospital, Montreal, QC, Canada

**Keywords:** synapse, excitation, plasticity, electrophysiology, microscopy, optogenetics

## Introduction

The development and function of the human brain as well as its remarkable capacity for experience dependent change hinge on the organization and dynamics of synapses. In the central nervous system, excitatory synapses represent the primary means of information processing by local circuits and communication between brain regions. The molecular composition, structural organization, signaling function, and plasticity of excitatory synapses underlie experience-dependent changes in brain function associated with learning and memory. Not surprisingly, disruption of excitatory synapse signaling, function, and plasticity has been implicated in a broad range of neurological and psychiatric diseases, including schizophrenia, autism, depression, substance abuse and addiction, Parkinson's disease, Alzheimer's disease, traumatic brain injury, stroke, and epilepsy. Therefore, synaptic studies not only provide fundamental insight into a linchpin of the nervous system but also is essential to develop novel therapeutics and progress in lessening the burden of human neurological diseases and improving mental health.

In the past decade, major progress has been made in understanding the architecture and functionalities of excitatory synapses. These advances, largely triggered by the advent of novel technologies—such as cryo-electron and super-resolution microscopy, optogenetics and opto-pharmacology, deep sequencing, single-cell genetics, etc. —have had profound implications for our understanding of the normal and diseased brain. In this Research Topic, we have collected articles to highlight recent progress and excitement across the breadth of synapse biology, with a focus on glutamatergic synapses of the mammalian brain and an emphasis on molecular, cellular, physiological, and physiopathological mechanisms.

## Papers in This Collection

### Synapse Development and Neurodevelopmental Disorders

Understanding how brain circuits form and how proper synaptic connectivity is established during brain development is an active area of neuroscience research that is essential for clarifying the mechanisms of neurodevelopmental disorders such as autism and intellectual disability. To study synapse development, culture methods are invaluable, as these provide direct access to these processes. In this collection, Moutin et al. detail a method for making postnatal cultures of mouse hippocampal neurons and for efficient genetic manipulation using lentiviruses. Because most excitatory synapse development in hippocampus occurs after birth, this method provides incredible access for imaging, functional studies, as well as for genetic and chronic pharmacological treatments to study synapse development. Lentiviral transfection also allows manipulation of neurodevelopment-related genes, to determine acute and direct effects of these genes on synapses.

The interaction between GABAergic and glutamatergic synapses during brain development and how they impact each other's maturation has been unclear. Using organotypic hippocampal slice cultures, Salmon et al. discovered a role for depolarizing GABA in limiting excitatory synapse development. Over the course of neuron development, GABA receptor (GABAR) mediated synaptic currents switch from depolarizing to hyperpolarizing after the first postnatal week, due to a developmental expression of the KCC2 chloride transporter. Salmon et al. demonstrated a developmental switch in GABAR current polarity in slice cultures and find that pharmacological blockade of GABARs during the first postnatal week, even transiently, induces a persistent increase excitatory synapse number. They also demonstrate that this effect requires action potential firing of neurons, suggesting that it is the depolarizing action of GABARs that restrains excitatory synapse development. This activity-dependent inhibition of synapse development may utilize similar mechanisms to the activity-induced synaptic depression or elimination mechanisms that occur in the brain.

Loss of the fragile X mental retardation protein FMRP is a leading monogenic cause of autism, called Fragile X Syndrome (FXS). FXS is also characterized by repetitive, stereotyped behaviors and hyperactivity—all of which are associated with striatal function. FMRP regulates structural and functional synapse development and plasticity in the neocortex and hippocampus, but little is known of FMRP function in the striatum. Using cortical-striatal co-culture Huebschman et al. find that medium-spiny neurons (MSNs) without FMRP, have deficits in of excitatory synapse development, as measured by colocalized PSD-95 puncta at synapses and dendritic spines. Surprisingly, re- or overexpression of FMRP also suppressed synapse development, suggesting a *U*-shaped effect of FMRP on synapse development. Using different mutants of FMRP, some implicated in FXS, Huebschman et al. reveal roles for specific RNA binding domains of FMRP in development of cortical striatal synapses.

A fascinating review article by Nguyen et al. discusses recent literature on the mutations in the Neuroligin (*Nlgn1-4*) gene family of postsynaptic cell adhesion molecules and the interactions of distinct family members on synapse development and implications for diseases such as autism. NLGNs, generally, promote synapse development and stabilization and NLGN3 and NLGN4 mutations are linked to autism. Nguyen et al. review recent work that revealed distinct protein sequences, trafficking and functions for NLGN4 on synapses when expressed on the X (NLGN4X) or Y (NLGN4Y) chromosomes that may help to explain the male bias of autism diagnoses with NLGN mutations.

### Plasticity of Excitatory Synapses, AMPA Receptors, and Disease

A major mechanism of plasticity of excitatory synapses, both during development and in the mature brain, is trafficking of the AMPA receptor (AMPAR) subtype of ionotropic glutamate receptors. Synaptic strength changes during long-term potentiation (LTP) and long-term depression (LTD) can occur by insertion or removal of GluA2 lacking AMPARs at synapses, in part because they have a higher conductance. GluA2-lacking AMPARs are typically GluA1 homomers and are Ca^2+^ permeable. Little has been known of the molecular mechanisms that control trafficking of GluA2- lacking, Ca^2+^ permeable AMPARs. Purkey and Dell'Acqua provide a comprehensive review of how phosphorylation and dephosphorylation of GluA1 regulates trafficking of Ca^2+^ permeable AMPARs at synapses during plasticity. The authors also provide important knowledge of the molecular mechanisms of synaptic plasticity that contribute to learning and memory.

Long-term synaptic plasticity and memory storage in the brain are well-known for depending on neuromodulation. Precisely how remains poorly understood, however. In a modeling study, Mihalas et al. explore how the expression of LTP and LTD is limited by the occupancy of AMPARs at small perisynaptic compartments, and how this is modulated by push-pull regulation by the G-proteins Gs and Gq. In addition to showing how their model captures the key features of the pull-push neuromodulation of synaptic plasticity, Mihalas et al. also demonstrate that it is consistent with other actions of neuromodulators observed in slice, for a view that is compatible with our current understanding of AMPAR trafficking in synaptic plasticity.

Caffeine modulates synaptic function through its antagonistic action at adenosine receptors, but little has been known of the age- and brain-region-specific functions of adenosine at synapses. New work from Caruana and Dudek reveals that activation of adenosine receptors induces a long-lasting depression of synaptic transmission at hippocampal CA1 and CA2 neurons in young rats, but selectively induces depression in CA2 in adults. They also find distinct sensitivity of CA1 and CA2 synaptic transmission to regulators of cAMP levels, a downstream effector of adenosine, which provides mechanistic insight into the CA2-specific actions of adenosine.

A wide range of neuropathologies, including psychiatric and neurodegenerative disorders, have aberrations in synaptic transmission and plasticity that are specific to brain region and to AMPAR subunits. In an interesting mini review, Zhang and Bramham provide a summary of AMPAR dysregulation in animal models of neuropathology. They also discuss preclinical findings regarding the targeting of AMPARs therapeutically.

Epileptogenesis is the process by which the healthy brain becomes epileptic. This process is not well-understood, to a large extent because of a limited availability of relevant probes to monitor the key players in epileptogenesis, for example the gelatinases Matrix Metalloproteinase 2 and 9 (MMP-2 and MMP-9, respectively). To address this, Bouquier et al. created a peptide-based biosensor for reporting gelatinase activity. As a proof of principle, they applied their new biosensor to the kainate model of epilepsy. Bouquier et al. propose that this biosensor is useful for localizing cellular reactive changes in epileptogenesis and that it could enable selective, local therapy by pharmacologically targeting gelatinase activity.

### Neuronal and Synaptic Heterogeneity in Humans and Rodents

There is much heterogeneity of neuronal and synaptic morphology and function in the brain, but little is known of this heterogeneity in human brain and how it relates to the evolution of cortical areas. In a hypothesis-and-theory article, Rasia-Filho et al. present new data and discuss previous work from 3D reconstruction of pyramidal neurons that demonstrate a morphological heterogeneity across the subcortical, allocortical, and neocortical regions of the human brain, focusing on the amygdala, hippocampus, and neocortex. They also demonstrate and discuss the large variability in dendritic spine morphologies within an individual pyramidal neuron in each region and discuss the functional implications of such diversity.

The cingulate cortex of humans has been implicated in emotion, attention, cognition, and social behavior. Interestingly, in layer 5 of the human cingulate cortex, one does not only find the classical pyramidal neurons, but also a specialized neuronal type with an elongated spindle-shaped cell body, known as the von Economo neuron, or VEN. Using advanced histological techniques, Correa-Júnior et al. studied these intriguing VENs in tissue from four neurologically normal adult subjects, which revealed a continuum of morphological properties. Morphometry suggested to Correa-Júnior et al. that this spectrum could be subdivided into three classes, VENs 1 through 3, with progressively increased dendritic ramifications and higher spine densities. The authors suggest that this interesting heterogeneity may underlie diverse functionality and forms of information processing.

Synapses in the brain are strikingly diverse. Even within a relatively restricted portion of the brain, such as the CA1 region of the hippocampus, there is a high degree of synaptic variability due to molecular and morphological differences present among individual synapses of the same type. However, most of today's electrophysiological methods record from synapse populations, thus averaging across individual synapses while discarding the individual variability. In a thought-provoking hypothesis-and-theory article, Grant and Fransén discuss the implications of synapse diversity for studies of synaptic physiology, plasticity, development, and behavior, as well as for phenotypes arising from pharmacological and genetic perturbations. Grant and Fransén propose that present models that are based on measurements averaged across diverse populations may need to be re-examined, since single-synapse resolution methods are required to test and validate current synaptic models of behavior. This fresh view suggests that novel, more detailed models of physiology and behavior could be created based on this traditionally ignored functional and molecular diversity of different synapses.

## Concluding Remarks

This collection highlights the varied and latest research concerning excitatory synapses. These works describe methods used to study synapses, as well as current efforts to understand the development, plasticity and function of excitatory synapses in both humans and animals. Importantly, several articles illustrate how research in excitatory synapses contributes to the understanding and treatment of brain disease. We hope this collection will inform and inspire future studies in many areas of neuroscience.

## Author Contributions

KH, PP, and PJS wrote the manuscript. All authors contributed to the article and approved the submitted version.

## Conflict of Interest

The authors declare that the research was conducted in the absence of any commercial or financial relationships that could be construed as a potential conflict of interest.

## Publisher's Note

All claims expressed in this article are solely those of the authors and do not necessarily represent those of their affiliated organizations, or those of the publisher, the editors and the reviewers. Any product that may be evaluated in this article, or claim that may be made by its manufacturer, is not guaranteed or endorsed by the publisher.

